# Abnormal Development of Dendrites in Adult-Born Rat Hippocampal Granule Cells Induced by Cyclophosphamide

**DOI:** 10.3389/fncel.2017.00171

**Published:** 2017-06-21

**Authors:** Lin Wu, Dandan Guo, Qi Liu, Fei Gao, Xiaochen Wang, Xueying Song, Fuwu Wang, Ren-Zhi Zhan

**Affiliations:** ^1^Department of Physiology, Shandong University School of MedicineJinan, China; ^2^Department of Histology and Embryology, Shandong University School of MedicineJinan, China

**Keywords:** adult neurogenesis, chemotherapy, cognition, dendrite, granule cell, hippocampus, spine

## Abstract

Although development of cognitive decline in cancer patients who receive chemotherapy is common, the underlying mechanism(s) remains to be identified. As abnormalities in adult hippocampal neurogenesis may serve as substrate for cognitive dysfunction, the present study examines the effect of cyclophosphamide (CPP), a widely prescribed chemotherapeutic agent, on dendritic development of adult-born hippocampal granule cells in the rat. CPP was intraperitoneally injected into male Sprague-Dawley rats once a week for four consecutive weeks. Four weeks and 1 week after the last dose of CPP, Morris water maze test and doublecortin (DCX) immunohistochemistry were carried out to determine the effects of CPP on cognitive function and the rate of hippocampal neurogenesis, respectively. Adult newborn hippocampal granule cells were labeled at the same day as the first dose of CPP and were examined 10 weeks after labeling. Results showed that cognitive decline induced by CPP was associated with both suppressed adult hippocampal neurogenesis and abnormal development of dendrites of newborn granule cells. The abnormalities of dendrites in newborn granule cells after CPP exposure included less dendritic branching, shorter total dendritic length, thinner and torturous dendritic shafts with intermittent appearances of varicosities, and lower spine densities of stubby and thin types along dendritic shafts, but an increased density of mushroom-like spines. Adult-born granule cells in the presence of CPP, a widely used anti-cancer medication, display abnormal dendritic morphologies and fewer dendritic spines which may underlie cognitive dysfunction.

## Introduction

Systemic cancer treatment is associated with cognitive decline that manifests as impairments in learning, memory formation, attention, information processing speed and executive functioning (Anderson-Hanley et al., 2003; Minisini et al., 2004; Vardy and Tannock, 2007; Janelsins et al., 2011; Dietrich et al., 2015; Iyer et al., 2015; Ono et al., 2015; Wefel et al., 2015). Multiple mechanisms including biological and neuropsychiatric responses to cancers (Andreotti et al., 2015; Wefel et al., 2015), toxic effects of chemotherapeutic agents (Ahles and Saykin, 2007; Hutchinson et al., 2012; Dietrich et al., 2015) and side effects of adjuvant hormones (Rugo and Ahles, 2003; Castellon et al., 2004) have been proposed for the development of cognitive dysfunction. Although both direct and indirect detrimental effects of chemotherapeutic agents on brain cells may contribute to chemotherapy-induced cognitive dysfunction, specific pathways have yet to be identified (Ahles and Saykin, 2007; Dietrich et al., 2015).

As a subcortical structure, the hippocampus functions in learning, memory formation and mood regulation. Granule cells in the hippocampal dentate gyrus are continuously to be generated throughout life in this brain region from neural stem/progenitor cells that reside in the subgranular zone. Ultimately, nearly a quarter of newborn granule cells survive to become mature neurons under normal conditions (Christian et al., 2014). Studies have shown that newborn granule cells integrate into the pre-existing dentate network and participate in almost all of the known functions of the hippocampus (Clelland et al., 2009; Sahay et al., 2011; Aimone et al., 2014; Christian et al., 2014; Opendak and Gould, 2015). The rate of adult hippocampal neurogenesis is under intense regulatory control of internal and external environments (Aimone et al., 2014). Studying in animals has shown that cyclophosphamide (CPP), a commonly used chemotherapeutic agent, suppresses adult hippocampal neurogenesis (Yang et al., 2010; Briones and Woods, 2011; Lyons et al., 2011; Christie et al., 2012; Dietrich et al., 2015). Given the fact that it takes 6~8 weeks for newborn granule cells to mature (Aimone et al., 2014; Christian et al., 2014; Bergami et al., 2015), a better understanding of detrimental effects of chemotherapeutic agents on cognitive function should focus not only on the rate of adult neurogenesis but also on the integration of newborn granule cells. The normal integration process needs newborn granule cells to be positioned appropriately, to have fully developed dendrites, and to innervate onto right targets. Maturation of newborn granule cells may be delayed by reduced physical or exploring activities (Ambrogini et al., 2010) which are present during and even after chemotherapy (Irwin et al., 2004). In the present study, we explored possible abnormalities of dendritic morphologies of newborn granule cells of the rat hippocampus after exposure to CPP.

## Materials and Methods

### Animals

All animal experiments were approved by the Animal Ethics Committee of Shandong University School of Medicine and performed according to the guides for the care and use of laboratory animals set by National Research Council (US). Male Sprague-Dawley rats (160–180 g, 6–8 weeks old) purchased from Beijing HFK Bioscience Company (Beijing, China) were housed in a university animal facility with a 12 h light/dark cycle and free access to food and water, and were weighed weekly before and during the experiments. In total, 34 animals were used in the present study; two animals died after CPP monohydrate (CPP) injection (all were in the 3rd week after initiation of injection). Three sets of experiments were conducted in 32 animals: 14 for Morris water maze testing, six for assessment of adult neurogenesis with doublecortin (DCX) immunohistochemistry and 12 for dendritic measurements following intradentate retroviral vector injection.

### Doses and Delivery of CPP

CPP monohydrate (Sigma, St. Louis, MO, USA) was dissolved in sterile saline to make a 50 mg/ml of stock solution. The stock solution was stored at −80°C until use. CPP was intraperitoneally injected into animals once a week for four consecutive weeks at either 25 mg/kg or 50 mg/kg. Animals received equivalent amount of saline in a schedule same to CPP-treated ones were used as controls.

### Transcardial Perfusion and Tissue Preparation

Each animal was deeply anesthetized with an intraperitoneal injection of sodium pentobarbital (80 mg/kg). After complete paralysis, the animal was perfused through the aorta with 50 ml heparinized saline, followed by 300 ml of 4% paraformaldehyde in 0.1 M phosphate-buffered saline (PBS, pH 6.8) over a period of 40 min. The brain was removed from the skull upon completion of perfusion, and then post-fixed in the same fixative at 4°C overnight. Thereafter, the brain was sequentially immersed in 10% sucrose in 0.1 M PBS for 4 h, 15% sucrose in 0.1 M PBS for 8 h, and finally 20% sucrose in 0.1 M PBS at 4°C overnight. Tissue block including the hippocampus was made with the aid of an appropriate brain matrix (WPI, Sarosata, FI, USA). After the tissue block was firmly embedded with a medium that consisted of 30% (w/v) chicken egg albumin (Sigma, St. Louis, MO, USA), 0.5% (w/v) gelatin, and 0.9% (v/v) glutaraldehyde in 0.1 M PBS as described previously (Zhan and Nadler, 2009), coronal sections were cut into 40 μm or 110 μm in thickness (depending on experimental aims) with a vibratome (VT1000 S, Leica Biosystems, Wetzlar, Germany).

### Doublecortin (DCX) Immunohistochemistry

The effect of CPP on the rate of neurogenesis was compared in saline- and CPP-injected rats (three animals each group) by staining and counting DCX-positive newborn granule cells. Brain sections were prepared 1 week after the final dose of CPP. Sections (40 μm in thickness) localized between 3.0 mm and 3.6 mm posterior to the bregma were serially cut. Sections with the order of number 3, 9 and 15 were subjected to DCX immunohistochemical staining with the free-floating procedure. After the collected sections were washed with 0.1 M PBS, they were incubated in a solution that consisted of 30% methanol and 3% H_2_O_2_ in water for 30 min to inactivate endogenous peroxidase. After rinsing, sections were incubated in a blocking solution that consisted of 2.5% BSA (Sigma, St. Louis, MO, USA), 0.2% Triton X-100 and 5% normal donkey serum in 0.1 M PBS at 4°C for 2.5 h to minimize non-specific reactions. Without rinsing, the sections were then incubated with a goat anti-DCX (Santa Cruz, San Jose, CA, USA) that was diluted with the blocking solution (1:400) at 4°C overnight. After three washes with 0.1 M PBS, the sections were incubated with biotinylated donkey anti-goat IgG (Jackson ImmunoResearch, West Grove, PA, USA) in a dilution of 1:400 at 4°C for 1.5 h. After rinsing with 0.1 M PBS, the sections were incubated with the avidin-peroxidase complex (Vector Labs., Burlingame, CA, USA) at 4°C for 60 min. After several washes with 0.1 M PBS, immunoreactions were visualized by an incubation with 3,3-diaminobenzidine, enhanced by the addition of 0.08% ammonium nickel sulfate (Vector Labs., Burlingame, CA, USA). The reaction was terminated by incubating the section with water. Sections were then flatly mounted on slide glasses and left to be air-dried for at least an hour. After dehydration with a series of graded ethanol solutions, the sections were then cleared in xylene and coverslipped with neutral balsam. DCX-positive cells in the dentate gyrus were counted and the lengths of the superior and inferior pyramidal blade of the dentate gyrus were measured in each section by using Image-Pro-Plus (Media Cybernetics, Rockville, MD, USA). The density of DCX-positive cells was calculated by dividing the number of cells with the total length of granule cell layer and expressed as the number of cells/mm. Values in three sections each animal were averaged.

### Behavioral Assessments

For animals to be used for behavioral studies, open field test assessing general locomotor activity was conducted to qualify animals before CPP exposure. To this aim, a 90 cm × 90 cm white board, divided into 36 equal squares by red lines was used as a field apparatus. Each side of the board was fenced to prevent animals to jump out. Animals were individually placed in the central square and allowed to explore the area freely. The numbers of crossing (squares crossed), rearing (standing on hind limbs), grooming (washing face) and urination were recorded for 5 min. After each trial, the apparatus was cleaned with ethanol. Qualified rats were randomly divided into two groups, receiving intraperitoneal injection of saline (control group) or CPP (CPP group); both were carried out once a week for a consecutive 4 weeks. Four weeks after the last injection, the Morris water maze was conducted to assess animals’ spatial learning and memory abilities. The water maze task was carried out for seven consecutive days in which the first 6 days were used to train the animals to find the hidden platform, whereas the last day was used to probe how well the animals remembered the position of the platform. A cylindrical pool (120 cm in diameter), filled with water (22–25°C) was placed in a quiet testing room. The cylindrical pool was equally divided into four quadrants with a removable platform (10 cm in diameter) to be located in the center of the first quadrant. The platform was set 2 cm below the surface of water to make the platform invisible to animals. A digital camera was mounted above the pool and linked to an automatic tracking system (SMART polyvalent video-tracking system, Panlab, Spain) to track and record the performance of individual animals. Several bright-colored cues posted on the wall of the maze were used to help animals to find the platform. In the training sessions, each rat was put into the pool from the four different quadrants with its head facing the wall to prevent the animal seeing the platform directly. If a rat could find the platform within 60 s, it would be allowed to stay onto the platform for 20 s before removal from the pool. Instead, if a rat failed to find the platform within 60 s, it would be guided onto the platform. The time taken to find the hidden platform (latency to escape) was recorded. On the 7th day, the platform was removed and each rat was put into the pool from a start point (the opposite quadrant of the first quadrant) to probe its memory. The rat was allowed to swim freely in the pool for 60 s and the path of navigation was traced. Swimming speed and the time spent in the target quadrant and non-target quadrants were collected.

### Construction of CAG–GFP Retroviral Vector

Concentrated CAG–GFP retroviral vector was made from an established stable virus-producing platinum-E cell line (Gao et al., 2015) through two-step ultraspeed centrifugation detailed below. After the frozen cells were thawed quickly in a 37°C water bath, 1 ml of cell suspension was immediately transferred into a 10 ml tube that contained 4 ml of Dulbecco’s modified eagle’s medium (DMEM). The cell suspension mixed with DMEM was centrifuged at 1000 rpm for 5 min. After discarding the supernatant, the cell pellet was re-suspended with 2 ml of M10 (10% serum-contained DMEM). The resulted cell suspension was transferred into a 10 ml culture dish in which 8 ml culture medium was pre-added. After mixing, cells were cultured in a 5% CO_2_ incubator at 37°C. Upon ~90% confluence was reached, culture medium was removed and the cells were rinsed twice with autoclaved PBS. After cells were digested in 1 ml of 0.25% trypsin-contained solution for 3–4 min, 4 ml of M10 was added into the dish. After thorough mixing, 2 ml of cell suspension was added into an 8 ml M10-contained 10 ml culture dish. At this stage, 12 dishes were prepared each time. Dishes were cultured in a 5% CO_2_ incubator at 37°C and the supernatant was collected 24 and 48 h after culture. Supernatants collected from all dishes were pooled together and centrifuged (18,000 rpm) at 4°C for 2 h. The pellet was suspended with 1 ml Dulbecco’s phosphate-buffered saline and then centrifuged (12,000 rpm) at 4°C for 1.5 h. After removal of supernatant, the pellet was suspended in 30 μl of Dulbecco’s phosphate-buffered saline. After being kept at 4°C overnight, the viral suspension was gently mixed, divided into 5–10 μl aliquots, and kept at −80°C until use.

### Intradentate Injection of CAG–GFP Retroviral Vector

After an animal was anesthetized with an intraperitoneal injection of sodium pentobarbital (25 mg/kg) and ketamine (60 mg/kg), the animal was placed on a stereotaxic frame suitable for rats (Stoelting, Wood Dale, IL, USA). After the skin was sterilized with a sequential application of 7.5% povidone iodine and 75% alcohol, the scalp was incised and a skull hole on the right side was drilled. One microliter of CAG–GFP retroviral vector was injected into the right dentate gyrus at a rate of 0.1 μl/min using a 1 μl Hamilton syringe (Bonaduz, GR, Switzerland) that was connected to an automatic injection pump (Stoelting, Wood Dale, IL, USA). The injection site was located 3.2 mm posterior from the bregma and 2.5 mm right to the midline, in a depth of 2.4 mm (measured from the surface of the cortex). The needle was left in the place for additional 15 min after the completion of injection to prevent possible backflow before removal. All surgical procedures were performed under sterile conditions. Animals were returned to cages after recovery from anesthesia.

### Enhancement of GFP Fluorescence with Anti-GFP Antibody for Dendritic Analyses

For dendritic measurements, transcardial perfusion was performed 10 weeks after retroviral vector injection. Coronally cut hippocampal sections (110 μm in thickness) in which GFP-labeled granule cell(s) presented were subjected to GFP immunostaining by the free-floating procedure. Sections were first washed with 1XPBS, and then incubated in a blocking solution that consisted of 2.5% BSA, 0.2% Triton X-100 and 5% goat serum in 1XPBS to minimize non-specific reactions. Thereafter, sections were incubated with a mouse anti-GFP (Invitrogen, Carlsbad, CA, USA) in a dilution of 1:1000 at 4°C overnight. After three washes with 1XPBS, the sections were incubated with an Alexa fluor 488-conjugated secondary antibody (Invitrogen, Carlsbad, CA, USA) in a dilution of 1:600. After another three washes with 1XPBS, sections were mounted on glass slides with 75% glycerol in PBS and coverslipped. Sections in which GFP was immunofluorescently amplified were scanned with the Zeiss 780 laser scanning microscope (Carl Zeiss, Jena, Germany). Images yielded were used for quantitative measurements of dendrite length, the complexity of dendritic arborization and dendrite spine.

### Measurements of Dendrite Length and Complexity

To measure dendrite length and the complexity of dendritic arborization, individual GFP-labeled cells residing in the suprapyramidal blade were z-stacked under a 20× objective with a z-series interval of 1 μm and zoom 1. A 3-dimensional image created from the z-series file each cell was compressed into a 2-demnesional image. Only cells with relatively intact dendritic arborizations were quantitatively analyzed further. After GFP-positive soma and dendritic arborization were manually traced under ImageJ^1^ with the NeuronJ plugin^2^, the length of each dendritic segment was measured. Total dendrite length was calculated by summing the lengths of all segments. After tracing, the complexity of dendritic arborization for individual cells was carried out by using Sholl analysis with the “Sholl plugin”. For Sholl analysis, the interval between concentric circles was 25 μm with the center point at the center of soma. For each rat, 3 cells were randomly picked for either of analysis and the values were averaged to represent the animal.

### Quantification of Dendritic Spines

z-stacks for dendritic segments located in the middle molecular layer were carried by using a 63× oil objective. The other parameters set for z-stacks were as follows: 0.12 μm of z-series thickness, zoom 4, and a spatial resolution of 0.03 μm × 0.03 μm × 0.12 μm. A 3-dimensional image was reconstructed from individual z-stack file. Spines were classified into stubby, thin or mushroom type based on spine length, head diameter to neck width ratio, and the size of head specific to dentate granule cells (Tyler and Pozzo-Miller, 2003; Boda et al., 2004; Zhao et al., 2014). A stubby spine had a length that was similar to the diameter of the neck as well as the diameter of the head. A mushroom spine displayed a great head diameter to neck width ratio in addition to a head surface area that was ≥0.4 μm^2^. The head surface area was estimated by using the function 0.25 × π × d_x_ (μm) × d_y_ (in μm); d_x_ and d_y_ were the lengths of shortest and longest axes of the spine head which were measured under ImageJ manually (Zhao et al., 2014). A spine that could not be classified into either stubby type or mushroom form with or without a bulbous head was referred to as the thin type. The numbers of total and spines in different forms on each segment were counted and the densities of total and individual types were calculated by dividing the numbers of spines with the length of corresponding dendritic segment. Six to nine dendritic segments (30–50 μm in length each) from at least three cells each animal located in the middle molecular layer were randomly scanned and analyzed. Total, stubby, thin and mushroom spine densities from all counted segments each animal were averaged and used to represent the animal.

### Statistical Analysis

Data are expressed as mean ± standard error of the mean (SEM). Two-group data are compared with unpaired Student’s *t*-test. One-way analysis of variance (ANOVA) was used to compare the means of three or more groups. Follow one-way ANOVA, Dunnett’s test was used for individual comparisons. Latency to escape was analyzed with repeated measures ANOVA, whereas data for Sholl analysis were compared with two-way ANOVA. A *p* ≤ 0.05 is considered to be significantly different.

## Results

### Long-Lasting Cognitive Decline after CPP Exposure Is Associated with a Suppression of Hippocampal Neurogenesis

**Figure 1:**
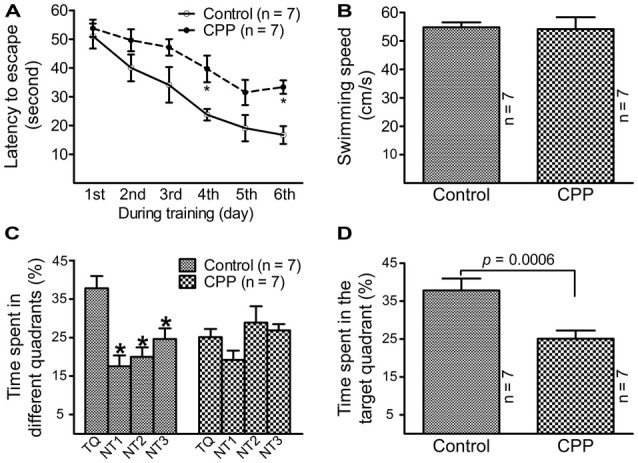
Reduced learning and memory abilities after cyclophosphamide (CPP) treatment revealed by Morris water maze test. Animals were tested 4 weeks after the last dose of CPP (50 mg/kg). Error bars are standard error of the mean (SEM). **(A)** Latency to escape recorded during the training days. *p < 0.01 (*t* = 2.82 at 4th and 2.99 at 6th, *df* = 72), in comparison with “control” at the same time revealed by by Dunnett *post hoc* test following repeated measures analysis of variance (ANOVA). **(B)** Swimming speed recorded in the testing day. **(C)** Time spent in the target quadrant in the testing day. *p < 0.05–0.01 (*df* = 24), in comparison with that in the target quadrant. Comparisons were done with Dunnett’s test following one-way ANOVA. TQ, NT1, NT2 and NT3 indicate the target quadrant, non-target quadrant 1, non-target quadrant 2 and non-target quadrant 3, respectively. **(D)** Time spent in the target quadrant between the control (Control) and CPP groups in the testing day (*t* = 2.81, *df* = 12).

To observe the effect of CPP on cognitive function, a dose of 50 mg/kg reported by a previous study (Christie et al., 2012) was applied. Before Morris water maze testing, body weight was measured and open field test was conducted in individual animals. Body weight was significantly lower in CPP group (379.0 ± 9.7 g, *n* = 7) in comparison with the control group (430.9 ± 4.8 g, *n* = 7; *p* < 0.005 via unpaired *t*-test). Open field test revealed that rats in CPP group (*n* = 7) had a trend to be less active than control group (*n* = 7). Events summed up from the number of crossing, rearing, glooming and urination in a 5 min-period was 51.3 ± 19.7 in CPP group and 107.0 ± 20.7 in the control group (*p* = 0.075, unpaired *t-test*). Morris water maze test revealed that 4-week CPP treatment significantly reduced learning and memory abilities, even though the test was held 4 weeks after the last dose of CPP. Times spent for finding the platform were analyzed with repeated measures ANOVA (Figure 1A). Since Chi-analysis revealed a violation of sphericity, data were analyzed with Huynh-Feldt correction. Statistical significances were found between the two groups (*F*_(1)_ = 22.9, *p* = 0.0004) and at different time points (*F*_(5)_ = 16.1, *p* < 0.0001) but no significant interaction between “group” and “time” (*F*_(5)_ = 0.84, *p* = 0.5247). At the first day of training, the time needed to find the platform (latency to escape) between the control (Control) and CPP groups was similar; however, as training days increased, animals in the control group spent less time finding the platform. A day after completion of training, the animals were tested on how firm they intended to find the platform for escape. As shown in Figure 1B, the swimming speeds were not statistically different between the control and CPP groups. However, animals in the control group stayed significantly longer in the target quadrant searching for the platform, whereas the same trend did not exist in the CPP-treated group (Figures 1C,D).

**Figure 2:**
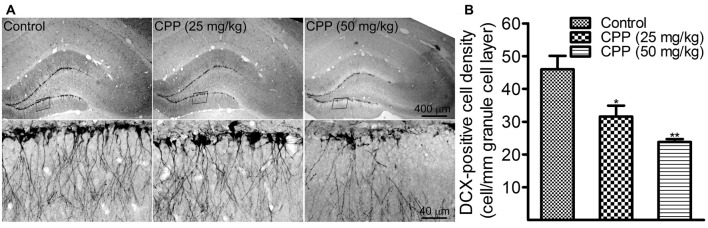
CPP reduces doublecortin (DCX)-positive cells in the hippocampal dentate gyrus. **(A)** Representative images showing DCX-positive cells in sections obtained from the control (left panel) and CPP-treated rats (middle and right panels). Upper row: imaged under a lower objective; Bottom row: areas indicated by the black boxes in the upper row were visualized under a higher magnification lens. **(B)** The densities of DCX-positive cells in the absence (Control) and the presence of two doses of CPP. The number of animals is three in each group. *p < 0.05 and **p < 0.01 (*df* = 6), in comparison with “Control” by Dunnett’s test following one-way ANOVA.

To determine if cognitive dysfunction induced by CPP is associated with a slower rate of adult hippocampal neurogenesis, we performed DCX immunohistochemistry. In addition to 50 mg/kg of CPP, a smaller dose was tested also. As shown in Figure 2A, at both doses, CPP significantly reduced the density of DCX-positive cells in the hippocampal dentate gyrus. The density of DCX-positive cells was reduced significantly from 46.0 ± 4.1 cells/mm in the control group (*n* = 3) to 31.2 ± 3.3 cells/mm in 25 mg/kg group (*n* = 3) and 23.8 ± 0.9 cells/mm in 50 mg/kg group (*n* = 3), respectively (Figure 2B).

### CPP Exposure Results in Less Dendritic Arborization and Complexity in Newborn Granule Cells

**Figure 3:**
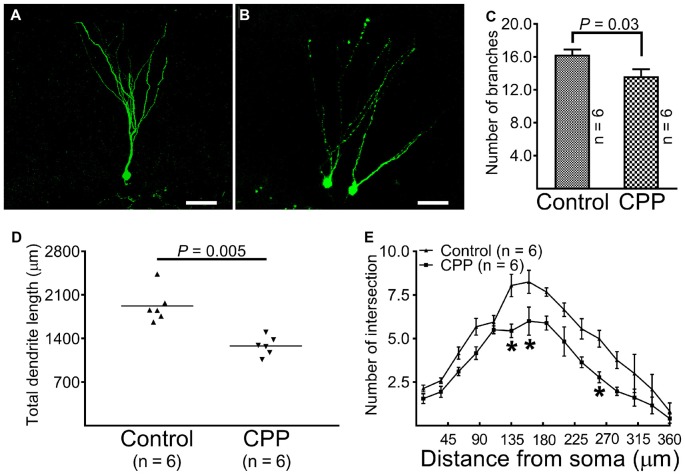
Representative images and statistical comparisons show the number of branch, total dendrite length and the complexity of arborization in the absence (Control) and presence of CPP. The first dose of CPP (50 mg/kg) was given at the same day as the CAG-GFP retroviral vector labeling and was continued for consecutive 4 weeks. Animals in the control group received equivalent amounts of saline. Animals in both groups were sacrificed 10 weeks after the injection of the retroviral vector. **(A)** A newborn granule cell obtained from a control rat (scale bar = 50 μm). **(B)** Newborn granule cells obtained from a rat treated with CPP (scale bar = 50 μm). **(C)** A comparison of the number of dendritic branches between the control (Control) and CPP groups. Each data point represents one animal in which the value was averaged from three newborn granule cells. Data are compared by unpaired student’s *t*-test. **(D)** A comparison of total dendrite length between the control (Control) and CPP groups. Each data point represents one animal in which the value was averaged from three newborn granule cells. Data are compared by unpaired student’s *t*-test. **(E)** Sholl analysis compares the complexity of dendritic arborization between the control (Control) and CPP groups. The numbers of animals studied are shown inside the figure. For each animal, the value was averaged from three cells. Statistical comparisons were done with two-way ANOVA. Sidak’s multiple comparisons were used for individual comparisons. *Indicates *p* < 0.05~0.001 as compared to the control (*df* = 150).

At 10 weeks after retroviral vector injection, GFP-labeled cells from control animals were found to reside in the border between the granule cell layer and the hilus with apical dendrites extending throughout the molecular layer and tips ending at the hippocampal fissure (Figure 3A). The morphology of dendritic tree of newborn granule cells obtained from CPP-treated animals appeared different from control rats in at least two aspects: they appeared to be beading (Figure 3B) and branched to a lesser extent (Figure 3C). Total dendrite length in the CPP group is 1279.0 ± 62.5 μm (*n* = 6), which is significantly shorter than 1921.0 ± 110.5 μm (*n* = 6) observed in the control group (*p* = 0.005, by unpaired *t*-test; Figure 3D). Sholl analysis revealed that dendritic arborization of newborn granule cells after CPP treatment is significantly less complex than that in the control group (*F*_(14,140)_ = 39.7, *p* < 0.0001), as shown in Figure 3E. Noticeably, the number of intersection indicating dendritic complexity was significantly less in CPP-treated group in dendritic segments (between 135 μm and 270 μm away from the soma).

### CPP Reduces Total Dendritic Spines but Increases Mushroom-Like Spines

**Figure 4:**
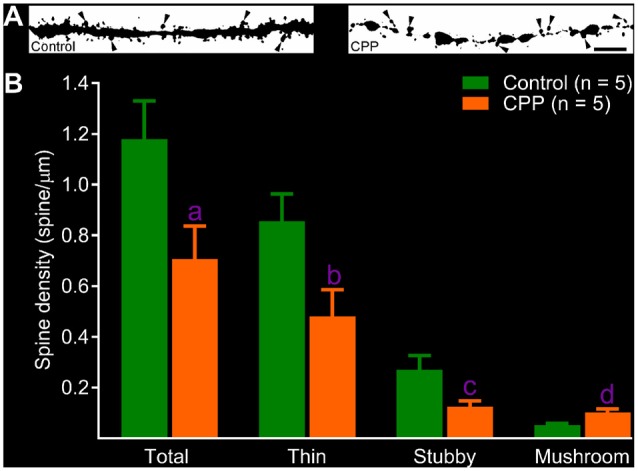
CPP reduces total but increases mushroom-like dendritic spines in adult-born granule cells. **(A)** Images of dendritic segments in 10 week-old newborn granule cells in a control animal (left panel) and a CPP (50 mg/kg)-treated animal (CPP, right panel). Mushroom-like spines are indicated by arrowheads. Scale bar = 5 μm. Note that dendritic segment obtained from a CPP-treated animal appears to be thinner with irregular varicosities (right panel). **(B)** Bar graphs show that CPP treatment reduces total, thin and stubby dendritic spines but increases mushroom-like spines. Error bars are SEMs. Statistical comparisons are done with unpaired *t*-test. “n” indicates the number of animals. “a”, “b”, “c” and “d” indicates *p* values at 0.045 (*t* = 2.37, *df* = 8), 0.038 (*t* = 2.48, *df* = 8), 0.043 (*t* = 2.41, *df* = 8) and 0.009 (*t* = 3.49, *df* = 8), respectively.

After revealing abnormalities in the dendritic tree of newborn granule cells after CPP treatment, we then quantified spines in dendritic segments of the middle molecular layer. In the control group, dense protrusions in variable shapes appeared along the relatively regular dendritic shaft (Figure 4A, left panel). CPP treatment resulted in unusual appearances of dendrites—shafts appeared to be thinner with intermittent varicosities, and in addition, fewer protrusions were present (Figure 4A, right panel). Total spine density in the control group (Control) is ~1.2 spines per μm length (*n* = 5), which was significantly denser than 0.7 spines per μm length (*n* = 5) in CPP-treated group (Figure 4B). In contrast to the density of total, stubby and thin spines, the density of mushroom-like spines, defined as having a ≥0.4 μm^2^ in head surface area with shorter neck, increased significantly in CPP-treated animals compared to the control animals (Figure 4B).

## Discussion

In addition to confirming that persistent cognitive decline induced by CPP is associated with a suppression of adult hippocampal neurogenesis, we found that CPP treatment leads to remarkable abnormal development of dendrites in newborn granule cells.

Multiple clinical surveys have provided solid evidence to show that chemotherapy can lead to cognitive decline, although the underlying mechanism remains to be fully elucidated (Anderson-Hanley et al., 2003; Minisini et al., 2004; Vardy and Tannock, 2007; Janelsins et al., 2011; Jim et al., 2012; Lindner et al., 2014; Dietrich et al., 2015; Wefel et al., 2015). Animal studies have found that chemotherapeutic agents, including CPP, profoundly slow the rate of adult hippocampal neurogenesis (Mustafa et al., 2008; Seigers et al., 2008, 2009, 2010; Yang et al., 2010; Briones and Woods, 2011; Lyons et al., 2011; Christie et al., 2012; ElBeltagy et al., 2012; Nokia et al., 2012). Because adult hippocampal neurogenesis is implicated in functions that overlap with those general functions of hippocampus (Clelland et al., 2009; Sahay et al., 2011; Aimone et al., 2014; Christian et al., 2014; Opendak and Gould, 2015), in addition to studying the rate of neurogenesis, studying the intrinsic electrophysiological properties and integration of newborn granule cells under the influence of chemotherapeutic agents is needed. For newborn granule cells to be integrated into the existing neural network properly, normal developments of dendrites and dendritic spines are essential. The integration process takes 6~8 weeks in mice (Aimone et al., 2014; Christian et al., 2014; Bergami et al., 2015), and it may be even longer in other species (Kohler et al., 2011), making this process potentially susceptible to environmental changes.

Clinical dosages of CPP vary according to the regimens of treatment. For intravenous injection, 40–50 mg/kg of CPP has been recommended for a consecutive 4 days (Cyclophosphamide FDA Packet Insert, 2013) in which several cycles of treatment may be given. Fifty milligram per kilogram of CPP was chosen for the examinations of cognitive functions and dendritic changes in the present study on the basis of the same dose which has been used in a previous study (Christie et al., 2012) and this dose resulted in nearly 50% reduction in DCX-labeled newborn granule cells (Figure 2). Since CPP was applied for a consecutive 4 weeks and dendritic morphological changes were examined more than 2 months after birth, dendritic changes observed in the present study are considered to be chronic response. To avoid the influence of intradentate retroviral vector injection on behavioral tests and behavioral training on dendritic developments, cognitive function, alteration in hippocampal neurogenesis and dendritic morphologies were not studied in the same set of animals rather than three separated sets of animals.

The Morris Water Maze was an established method for testing hippocampus-dependent learning, acquisition of spatial memory and long-term spatial memory. The substrate for those hippocampus-dependent cognitive deficits during and after CPP treatment has been firmly established. A recent study revealed that CPP treatment was associated with abnormal dendritic structures in hippocampal principal cells (Acharya et al., 2015). By combining retroviral labeling and confocal imaging, CPP was found to cause an abnormal development of dendrites in newborn granule cells. The characteristics of dendritic abnormality include fewer branches and shorter total length of dendrites, irregularity of dendritic shafts especially intermediate dendritic varicosities along the thinner and tortuous shafts, and sparser dendritic spines but increased mushroom-like spines. Because the morphology of dendrites and spines attached to dendritic shafts are critical in receiving excitatory projections and in cellular signal integration (Šišková et al., 2014), the abnormalities observed after CPP treatment are expected to result in lower excitability and insufficient integration of newborn granule cells, which could contribute to the development of cognitive decline. In comparison with dendrites of newborn granule cells in the controlled animals, dendrites of newborn granule cells in the presence of CPP appeared to be intermediately thinner and torturous. Since these features were not obvious in pre-existing granule cells in a similar treatment regimen (Acharya et al., 2015), dendritic changes in newborn granule cells are unlikely to be caused by direct toxic effect but developmental defect. While total spine density (due to decreased thin and stubby spines) was found to be lower in CPP-treated animals, the mushroom-like spine density was rather increased. As larger spines are generally considered to be composed by more excitatory amino acid receptors (Matsuzaki et al., 2001; Noguchi et al., 2005) and transformed from small spines in response to sensory stimuli (Grutzendler et al., 2002; Yasumatsu et al., 2008), the increase in mushroom-like spines after CPP treatment is puzzling. Several mechanisms may underlie the increased density in mushroom spines in the presence of CPP. First, it is possible that the increased density of mushroom-like spines after CPP exposure might have been a compensatory response, since persistent lack of excitatory synapses is known to result in increased head diameter and strength of remaining spines after naturation (Turrigiano, 2007). In addition, changes in dendritic geometry may alter integration of excitatory inputs, resulting in only few spines to be more active and consequently larger. Indeed, increases in mushroom-like dendritic spines have been observed in later stages of Alzheimer’s disease-like pathology (Dickstein et al., 2010) and in Fyn defect mice (Babus et al., 2011) as well. It remains unknown if direct damage to CA1 pyramidal cells by CPP might have affected the development of newborn granule cells.

The development of dendrites in newborn granule cells is under strong regulatory control of the internal and external environments (Aimone et al., 2014). It remains unclear what mechanism(s) may underlie abnormal development of new born granule cells after CPP exposure. BDNF, along with nerve growth factor (NGF), neurotrophin-3 (NT3) and neurotrophin-4/5 (NT4/5), belongs to a family of closely related, small, secreted proteins called neurotrophins. Mainly through acting on tyrosine receptor kinases, BDNF affects neuronal proliferation, differentiation, survival and morphological maintenance, as well as dendrite outgrowth (Waterhouse and Xu, 2009). Additionally, BDNF is known to be involved in numerous processes of functional and structural synaptic plasticity (Zagrebelsky and Korte, 2014). Evidence obtained from the hippocampal dentate gyrus shows that global over-expression of BDNF promotes growth of dendrites in dentate granule cells (Tolwani et al., 2002), while deficits in BDNF signaling lead to abnormal dendritic development of newborn granule cells (Bergami et al., 2008; Chan et al., 2008; Wang et al., 2015). In addition to dendritic morphology, it appears that following BDNF deficits, the dendritic spines display more dramatic and specific changes than dendrite morphology (Zagrebelsky and Korte, 2014). The features of dendritic abnormality after CPP treatment are very similar to those caused by BDNF deficits, which include decreased dendrite branching, length and complexity (Bergami et al., 2008; Chan et al., 2008; Wang et al., 2015). CPP-induced decrease in total spine density with an increase in mushroom spine density is also opposite to what has been observed after BDNF application (Tyler and Pozzo-Miller, 2003). It is reasonable to consider that the abnormal development of dendrites in newborn granule cells in the presence of CPP might have been at least partially mediated by the down-regulation of BDNF expression. However, future studies are needed to examine if endogenously generated or externally applied BDNF could rescue detrimental developmental changes caused by CPP.

In conclusion, adult-born granule cells in the presence of CPP, a widely used anti-cancer medication, display abnormal dendritic morphologies and fewer dendritic spines, which may relate to chemotherapeutic agents-induced cognitive dysfunction.

## Author Contributions

LW did most of animal experiments, behavioral studies and imaging data processing. DG and QL did immunohistochemistry and data analysis. FG, XS and XW did confocal imaging. FW helped in experimental design. R-ZZ designed the study and wrote the article.

## Conflict of Interest Statement

The authors declare that the research was conducted in the absence of any commercial or financial relationships that could be construed as a potential conflict of interest.

## Footnotes

^1^http://imagej.nih.gov/ij/

^2^http://www.imagescience.org/meijering/software/neuronj//
